# A Longitudinal Study of Association between Adiposity Markers and Intraocular Pressure: The Kangbuk Samsung Health Study

**DOI:** 10.1371/journal.pone.0146057

**Published:** 2016-01-05

**Authors:** Di Zhao, Myung Hun Kim, Roberto Pastor-Barriuso, Yoosoo Chang, Seungho Ryu, Yiyi Zhang, Sanjay Rampal, Hocheol Shin, Joon Mo Kim, David S. Friedman, Eliseo Guallar, Juhee Cho

**Affiliations:** 1 Department of Epidemiology and Welch Center for Prevention, Epidemiology, and Clinical Research, Johns Hopkins University Bloomberg School of Public Health, Baltimore, Maryland, United States of America; 2 Department of Epidemiology, Graduate School of Public Health, Seoul National University, Seoul, South Korea; 3 National Center for Epidemiology, Carlos III Institute of Health and Consortium for Biomedical Research in Epidemiology and Public Health (CIBERESP), Madrid, Spain; 4 Center for Cohort Studies, Total Healthcare Center, Kangbuk Samsung Hospital, Sungkyunkwan University School of Medicine. Seoul, South Korea; 5 Department of Occupational Medicine, Kangbuk Samsung Hospital, Sungkyunkwan University School of Medicine, Seoul, South Korea; 6 Department of Social and Preventive Medicine, Julius Centre University of Malaya, Faculty of Medicine, University of Malaya, Kuala Lumpur, Malaysia; 7 Department of Family Medicine, Kangbuk Samsung Hospital and Sungkyunkwan University School of Medicine, Seoul, South Korea; 8 Department of Ophthalmology, Kangbuk Samsung Hospital, Sungkyunkwan University School of Medicine, Seoul, South Korea; 9 Dana Center for Preventive Ophthalmology, The Wilmer Eye Institute, Johns Hopkins University, Baltimore, Maryland, United States of America; 10 Department of Health Sciences and Technology, SAIHST, Sungkyunkwan University, Seoul, South Korea; 11 Biostatistics and Clinical Epidemiology Center, Samsung Medical Center, Sungkyunkwan University School of Medicine Seoul, South Korea; Massachusetts Eye & Ear Infirmary, Harvard Medical School, UNITED STATES

## Abstract

**Importance:**

Intraocular pressure (IOP) reduction or stabilization is the only proven method for glaucoma management. Identifying risk factors for IOP is crucial to understand the pathophysiology of glaucoma.

**Objective:**

To examine the associations of change in body mass index (BMI), waist circumference, and percent fat mass with change in intraocular pressure (IOP) in a large sample of Korean adults.

**Design, setting and participants:**

Cohort study of 274,064 young and middle age Korean adults with normal fundoscopic findings who attended annual or biennial health exams from January 1, 2002 to Feb 28, 2010 (577,981 screening visits).

**Exposures:**

BMI, waist circumference, and percent fat mass.

**Main Outcome Measure(s):**

At each visit, IOP was measured in both eyes with automated noncontact tonometers.

**Results:**

In multivariable-adjusted models, the average increase in IOP (95% confidence intervals) over time per interquartile increase in BMI (1.26 kg/m^2^), waist circumference (6.20 cm), and percent fat mass (3.40%) were 0.18 mmHg (0.17 to 0.19), 0.27 mmHg (0.26 to 0.29), and 0.10 mmHg (0.09 to 0.11), respectively (all *P* < 0.001). The association was stronger in men compared to women (*P* < 0.001) and it was only slightly attenuated after including diabetes and hypertension as potential mediators in the model.

**Conclusions and Relevance:**

Increases in adiposity were significantly associated with an increase in IOP in a large cohort of Korean adults attending health screening visits, an association that was stronger for central obesity. Further research is needed to understand better the underlying mechanisms of this association, and to establish the role of weight gain in increasing IOP and the risk of glaucoma and its complications.

## Introduction

Intraocular pressure (IOP) is a key risk factor in the development and progression of primary open-angle glaucoma[1–3], and is associated with demographic factors including age, sex, race, as well as ocular and systemic factors such as central corneal thickness, refractive error, and anterior chamber anatomy.[4–6] IOP reduction or stabilization is the only proven method for glaucoma management, which may slow progression even for normal-tension glaucoma.[7–9] IOP is a complex trait influenced by multiple risk factors.[10–12] Identifying risk factors for IOP is crucial to understand the pathophysiology of glaucoma and to develop strategies that may prevent the onset or improve the prognosis of the disease.

Elevated IOP is associated with hypertension[13–17] and diabetes,[18–22] two obesity-related cardiovascular (CVD) risk factors. Increased body mass index (BMI) was associated with increased IOP in several longitudinal studies,[12, 23–28] but these studies were limited by small sample sizes, short follow-up period, or use of statistical methods that did not separate cross-sectional from longitudinal effects. Furthermore, all previous longitudinal studies used only BMI as a marker of adiposity, which may not reliably reflect overall or central adiposity.[29]

The objective of this study was thus to evaluate the longitudinal association between changes in adiposity markers including BMI, waist circumference and percent fat mass measured by impedance and IOP in a large cohort of adult Korean men and women attending regular health screening visits.

## Materials and Methods

### Study design and population

The Kangbuk Samsung Health Study is a longitudinal cohort study of Korean men and women 18 years of age or older who underwent comprehensive annual or biennial screening health examinations at the two Kangbuk Samsung Hospital Health Screening Centers in Seoul and Suwon, South Korea.[30] Health exams were scheduled every 2 years for participants younger than 40 years of age and every year for participants 40 years of age or older. Over 80% of the participants were employees of various companies or local government organizations and their spouses and the health screening exams paid by employers under the Korean Industrial Safety and Health Law. The remaining participants voluntarily purchased self-paid screening exams at the health screening center.

The present analysis included 281,238 study participants (completing 604,416 screening visits) who had tonometry as part of a health screening exam between January 1, 2002 and February 28, 2010. We excluded participants with missing IOP measurements at all visits (290 participants, 393 visits). We also excluded visits after participants developed an absolute difference in IOP between both eyes greater than 6 mm Hg, as this is atypical and may indicate secondary causes of IOP change[31] (9,225 visits in 4,090 participants); visits with missing fundus photograph (1,374 visits in 982 participants); and visits after participants developed abnormal findings in fundus photographs (15,443 visits in 8,967 participants). Since some exclusion criteria overlapped, the final study sample included 274,064 participants (119,723 women and 154,341 men, 97.4% of the total population) free of eye disease with a total of 577,981 screening visits. The average (SD) duration of follow-up was 1.8 (2.2) years and the average number of visits per participant was 2.1.

The Institutional Ethics Committee of the Kangbuk Samsung Hospital approved this study. The Ethics Committee waived the requirement of informed consent as we used only de-identified data routinely collected during health screening visits. This paper followed STOBE guidelines.[32]

### Measurements

At each screening exam, IOP was measured in both eyes with automated noncontact tonometers (2002–2004: TX-10, Canon, Tokyo, Japan; 2005–2008: TX-F, Topcon, Itabashi, Tokyo, Japan; 2009 onwards: CT-80, Topcon, Itabashi, Tokyo, Japan). Extreme IOP readings below 5 mm Hg (0.02%) or above 30 mm Hg (0.16%) were excluded from the analysis. The IOP measurement time was categorized as morning or afternoon. Fundus photographs were taken with a nonmydriatic fundus camera (CR6-45NM; Canon).

Height, weight, waist circumference and body composition were measured by trained nurses with participants wearing a light weight hospital gown and no shoes. BMI was calculated as weight in kilograms divided by height in meters squared. Waist circumference was measured at the midpoint between the bottom of the rib cage and above the top of the iliac crest to the nearest 0.1 cm while subjects were standing with their weight equally distributed on both feet, their arms at their sides, and head facing straight forward. Waist circumference was measured only in participants attending the Seoul center (n = 184,579). Percent fat mass was measured using a multi-frequency bioimpedance analyzer (Inbody 3.0 and Inbody 720, Biospace Co., Seoul, Korea).

Demographic characteristics, smoking status, alcohol consumption, physical activity, medical history, and medication use were collected through standardized, self-administered questionnaires. Smoking status was categorized into never, former, or current smoking; frequency of current alcohol consumption was categorized into < 1, 1–3, or > 3 days/week, and frequency of vigorous physical activity was categorized into none, 1–3, or > 3 times/week. Trained nurses measured sitting blood pressure and heart rate. Hypertension was defined as a systolic blood pressure ≥ 140 mm Hg, a diastolic blood pressure ≥ 90 mm Hg, a self-reported history of hypertension, or current use of antihypertensive medications.

Blood specimens were sampled from the antecubital vein after at least 12 hours of fasting. Serum glucose, total cholesterol, high-density lipoprotein (HDL) cholesterol, and triglycerides were measured as described elsewhere.[30] Diabetes mellitus was defined as a fasting serum glucose ≥ 126 mg/dL (≥ 7 mmol), a self-reported history of diabetes, or current use of antidiabetic medications.

### Statistical analysis

To assess the longitudinal association between changes in adiposity markers and IOP over time, as well as the cross-sectional association of adiposity markers with IOP at baseline, we used linear mixed models for longitudinal paired-eye IOP data.[33, 34] Details of the models are provided in the **Statistical Appendix**. Briefly, the models included separate fixed slopes for the baseline level and the change in adiposity markers from baseline to subsequent follow-up visits, and allowed for random variations in baseline IOP levels and longitudinal IOP slopes across participants and between eyes of the same participant. In these mixed models, the random effects accounted for correlations in IOP measurements arising from both paired eyes and repeated measurements over time, whereas the fixed effects provided estimates of the average longitudinal change in IOP per 1 interquartile difference in adiposity markers over time (corresponding to 1.26 kg/m^2^ for BMI, 6.20 cm for waist circumference, and 3.40% for percent fat mass) and the average cross-sectional difference in baseline IOP associated with 1 interquartile increase in baseline adiposity markers across participants (corresponding to 4.24 kg/m^2^ for BMI, 13.40 cm for waist circumference, and 8.81% for percent fat mass).

The models analyzed each body adiposity marker separately and were fitted with increasing degrees of adjustment. The first model adjusted for sex (male or female), study center (Seoul or Suwon), baseline age (continuous), and age change over time (continuous). The second model further adjusted for potential confounding effects of height (continuous) and baseline levels and changes over time in IOP measurement time (morning or afternoon), smoking status (never, former, or current), alcohol drinking (< 1, 1–3, or > 3 days/week), physical activity (none, 1–3, or > 3 times/week), and heart rate (continuous). The third model further included potential mediators of the effect of adiposity markers on IOP, such as baseline levels and changes over time in hypertension (yes or no) and diabetes (yes or no).

To allow for nonlinear longitudinal and cross-sectional relationships between adiposity markers and IOP, baseline levels and changes over time in adiposity markers were modeled using both quartile indicators and restricted quadratic splines with knots at the 5th, 50th, and 95th percentiles. Longitudinal and cross-sectional effect modifications by sex were also explored by including interactions between sex and restricted quadratic splines for baseline adiposity marker levels and changes over time.

We conducted sensitivity analyses without excluding participants with abnormal fundoscopy findings or with between-eye differences in IOP greater than 6 mmHg (280,911 participants with 604,416 visits). In addition, we restricted the analysis to participants with two or more screening visits (130,991 participants with 435,262 visits). All reported *P* values were two-sided and the significance level was set at 0.05. Statistical analyses were undertaken using Stata (version 12; Stata Corp., College Station, Texas).

## Results

The mean (SD) BMI, waist circumference, percent fat mass, and IOP of study participants at baseline were 23.5 (3.2) kg/m^2^, 79.8 (9.5) cm, 24.9 (6.4) % and 13.5 (2.7) mmHg, respectively (**Table 1**), with intraclass correlation coefficients over repeated visits of 0.94, 0.87, 0.90, and 0.66, respectively. Participants who had higher baseline IOP were younger, more likely to be males, smokers, alcohol drinkers, and had higher heart rate, blood pressure, cholesterol levels, fasting glucose, BMI, waist circumference and percent fat mass compared with participants with the lowest quartile of IOP levels.

**Table 1:** Baseline characteristics of study participants overall and by quartile of intraocular pressure.*

		Quartile of baseline intraocular pressure†, mmHg			
Characteristic	Overall	Quartile 1	Quartile 2	Quartile 3	Quartile 4
		(< 11.6)	(11.6–13.5)	(13.6–15.5)	(> 15.5)
Participants	273,522	71,206	77,036	68,777	56,503
Intraocular pressure†, mmHg	13.5 ± 2.7	10.3 ± 1.0	12.8 ± 0.6	14.7 ± 0.6	17.4 ± 1.4
Age, years	40.1 ± 10.0	40.2 ± 10.2	40.3 ± 10.1	40.1 ± 9.9	39.7 ± 9.8
Sex					
Male	153,964 (56.3)	31,521 (44.3)	42,788 (55.5)	43,042 (62.6)	36,613 (64.8)
Female	119,558 (43.7)	39,685 (55.7)	34,248 (44.5)	25,735 (37.4)	19,890 (35.2)
Study center					
Seoul	184,579 (67.5)	47,876 (67.2)	53,146 (69.0)	47,838 (69.6)	35,719 (63.2)
Suwon	88,943 (32.5)	23,330 (32.8)	23,890 (31.0)	20,939 (30.4)	20,784 (36.8)
Smoking status					
Never	146,797 (54.7)	43,791 (62.7)	41,828 (55.3)	34,131 (50.5)	27,047 (48.7)
Former	45,385 (16.9)	9,833 (14.1)	12,828 (17.0)	12,378 (18.3)	10,346 (18.6)
Current	76,411 (28.4)	16,212 (23.2)	20,985 (27.7)	21,111 (31.2)	18,103 (32.6)
Alcohol drinking, days/week					
< 1	172,507 (64.0)	49,438 (70.4)	49,177 (64.7)	41,129 (60.6)	32,763 (58.8)
1–3	70,964 (26.3)	15,563 (22.2)	19,570 (25.8)	19,241 (28.4)	16,590 (29.8)
> 3	26,231 (9.7)	5,210 (7.4)	7,206 (9.5)	7,490 (11.0)	6,325 (11.4)
Physical activity, times/week					
0	147,395 (54.6)	39,622 (56.4)	41,546 (54.6)	36,368 (53.5)	29,859 (53.5)
1–3	77,858 (28.8)	18,736 (26.7)	21,950 (28.8)	20,377 (30.0)	16,795 (30.1)
> 3	44,899 (16.6)	11,937 (17.0)	12,620 (16.6)	11,223 (16.5)	9,119 (16.4)
Height, cm	166.1 ± 8.6	165.2 ± 8.4	166.1 ± 8.6	166.7 ± 8.6	166.7 ± 8.6
Heart rate, beats/min	67.1 ± 9.3	65.6 ± 8.7	66.7 ± 9.0	67.7 ± 9.4	69.0 ± 10.0
Body mass index, kg/m^2^	23.5 ± 3.2	22.8 ± 3.0	23.4 ± 3.1	23.8 ± 3.2	24.2 ± 3.3
Waist circumference, cm	79.8 ± 9.5	77.4 ± 9.3	79.6 ± 9.4	81.0 ± 9.3	81.7 ± 9.4
Percent fat mass, %	24.9 ± 6.4	24.9 ± 6.4	24.8 ± 6.4	24.8 ± 6.4	25.3 ± 6.4
Diabetes	10,707 (3.9)	1,615 (2.3)	2,485 (3.2)	2,976 (4.3)	3,631 (6.4)
Fasting Glucose, mg/dL	95.2 ± 16.8	92.5 ± 12.5	94.5 ± 15.2	96.2 ± 17.4	98.7 ± 21.6
Total cholesterol, mg/dL	194.6 ± 35.2	189.8 ± 34.2	193.8 ± 34.8	196.4 ± 35.2	199.7 ± 36.1
HDL cholesterol, mg/dL	55.3 ± 12.4	56.2 ± 12.8	55.4 ± 12.4	54.9 ± 12.2	54.5 ± 12.1
LDL cholesterol, mg/dL	112.4 ± 29.8	108.8 ± 29.0	111.8 ± 29.5	113.8 ± 29.8	116.3 ± 30.5
Triglycerides, mg/dL	126.8 ± 86.1	110.7 ± 71.8	124.0 ± 82.6	133.6 ± 91.7	143.3 ± 96.6
Hypertension	47,494 (17.4)	8,247 (11.6)	12,161 (15.8)	13,343 (19.4)	13,743 (24.3)
Systolic blood pressure, mmHg	114.8 ± 14.6	111.2 ± 13.6	114.1 ± 14.0	116.3 ± 14.5	118.8 ± 15.3
Diastolic blood pressure, mmHg	74.3 ± 10.0	71.8 ± 9.5	73.9 ± 9.8	75.4 ± 10.0	76.9 ± 10.4

* Data are means ± SDs or number (%).

† Means (between-subject SDs) for the average intraocular pressure of left and right eyes.

**Fig 1:**
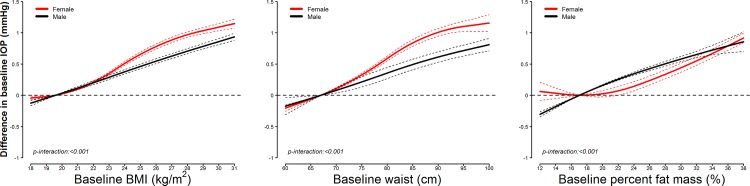
Smooth cross-sectional associations between adiposity markers and intraocular pressure at baseline. Curves represent average cross-sectional differences in baseline intraocular pressure (solid lines) and their 95% confidence intervals (dashed lines) based on restricted quadratic splines for baseline adiposity marker levels with knots at the 5th, 50th, and 95th percentiles. The reference value was set at the 10th percentile of each baseline adiposity marker. Results were obtained from linear mixed models with random variations in baseline intraocular pressure levels across participants and between eyes within participants, and adjusted for sex (male or female), study center (Seoul or Suwon), height (continuous), and baseline levels of age (continuous), intraocular pressure measurement time (morning or afternoon), smoking status (never, former, or current), alcohol drinking (< 1, 1–3, or > 3 days/week), physical activity (none, 1–3, or > 3 times/week), heart rate (continuous), hypertension (yes or no), and diabetes (yes or no). Histograms represent the frequency distributions of baseline adiposity markers.

**Fig 2:**
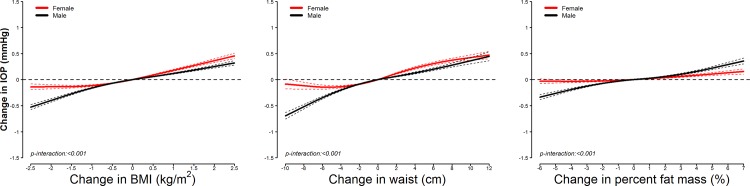
Smooth cross-sectional associations between adiposity markers and intraocular pressure at baseline, by sex. Curves represent average cross-sectional differences in baseline intraocular pressure (solid lines) and their 95% confidence intervals (dashed lines) based on restricted quadratic splines for baseline adiposity marker levels with knots at the 5th, 50th, and 95th percentiles. The reference value was set at the 10th percentile of each baseline adiposity marker. Results were obtained from linear mixed models with interactions between spline terms and sex, random variations in baseline intraocular pressure levels across participants and between eyes within participants, and adjusted for study center (Seoul or Suwon), height (continuous), and baseline levels of age (continuous), intraocular pressure measurement time (morning or afternoon), smoking status (never, former, or current), alcohol drinking (< 1, 1–3, or > 3 days/week), physical activity (none, 1–3, or > 3 times/week), heart rate (continuous), hypertension (yes or no), and diabetes (yes or no).

In cross-sectional analyses, baseline differences in adiposity markers across participants were positively associated with differences in IOP (**Table 2 and Fig 1**). In models adjusted for potential confounders (Model 2), the average baseline IOP increase associated with an interquartile baseline increase in BMI, waist circumference, and percent fat mass was 0.458 mmHg (95% confidence interval [CI] 0.444 to 0.472), 0.511 mmHg (0.490 to 0.533) and 0.497 mmHg (0.479 to 0.514), respectively. The cross-sectional associations were weaker in men than in women (**Fig 2**).

**Table 2:** Cross-sectional associations of adiposity markers with intraocular pressure at baseline.*

	Interquartile increase in baseline adiposity marker	Quartile of baseline adiposity marker				
		Quartile 1	Quartile 2	Quartile 3	Quartile 4	*P* value†
**Body mass index**						
Median baseline level (range), kg/m^2^	23.37 (13.66, 61.44)	19.93 (13.66, 21.27)	22.37 (21.27, 23.37)	24.37 (23.37, 25.51)	27.07 (25.51, 61.44)	
No. of subjects	274,041	68,513	68,508	68,510	68,510	
Adjusted mean difference in baseline intraocular pressure (95% CI), mmHg						
Unadjusted	0.601 (0.589, 0.614)	Reference	0.377 (0.351, 0.404)	0.713 (0.687, 0.740)	1.156 (1.129, 1.183)	< 0.001
Model 1‡	0.509 (0.496, 0.523)	Reference	0.275 (0.248, 0.303)	0.533 (0.504, 0.561)	0.951 (0.922, 0.980)	< 0.001
Model 2§	0.458 (0.444, 0.472)	Reference	0.257 (0.230, 0.284)	0.490 (0.462, 0.518)	0.860 (0.831, 0.889)	< 0.001
Model 3||	0.393 (0.379, 0.407)	Reference	0.249 (0.222, 0.276)	0.452 (0.424, 0.480)	0.744 (0.715, 0.774)	< 0.001
**Waist circumference**						
Median baseline level (range), cm	80.00 (36.00, 142.00)	68.60 (36.00, 73.00)	77.00 (73.10, 80.00)	83.20 (80.10, 86.40)	90.80 (86.50, 142.00)	
No. of subjects	159,888	42,421	39,421	38,225	39,821	
Adjusted mean difference in baseline intraocular pressure (95% CI), mmHg						
Unadjusted	0.615 (0.598, 0.632)	Reference	0.488 (0.454, 0.522)	0.820 (0.786, 0.854)	1.115 (1.082, 1.149)	< 0.001
Model 1‡	0.519 (0.498, 0.540)	Reference	0.392 (0.356, 0.427)	0.640 (0.600, 0.679)	0.902 (0.861, 0.943)	< 0.001
Model 2§	0.511 (0.490, 0.533)	Reference	0.381 (0.345, 0.417)	0.616 (0.576, 0.656)	0.885 (0.843, 0.927)	< 0.001
Model 3||	0.434 (0.412, 0.456)	Reference	0.362 (0.326, 0.398)	0.557 (0.517, 0.596)	0.754 (0.712, 0.796)	< 0.001
**Percent fat mass**						
Median baseline level (range), %	24.46 (1.25, 78.52)	17.79 (1.25, 20.44)	22.49 (20.44, 24.46)	26.61 (24.46, 29.25)	32.57 (29.25, 78.52)	
No. of subjects	255,050	63,768	63,764	63,758	63,760	
Adjusted mean difference in baseline intraocular pressure (95% CI), mmHg						
Unadjusted	0.092 (0.078, 0.106)	Reference	0.113 (0.085, 0.141)	0.052 (0.024, 0.080)	0.107 (0.079, 0.135)	< 0.001
Model 1‡	0.605 (0.588, 0.622)	Reference	0.341 (0.313, 0.369)	0.569 (0.540, 0.599)	1.024 (0.990, 1.057)	< 0.001
Model 2§	0.497 (0.479, 0.514)	Reference	0.274 (0.246, 0.302)	0.446 (0.416, 0.475)	0.825 (0.790, 0.859)	< 0.001
Model 3||	0.428 (0.410, 0.445)	Reference	0.226 (0.198, 0.254)	0.365 (0.335, 0.395)	0.703 (0.669, 0.738)	< 0.001

* Results were obtained from linear mixed models with random variations in baseline intraocular pressure levels across participants and between eyes within participants.

† *P* value for linear trend using an ordinal variable with the median baseline adiposity marker level in each quartile.

‡ Adjusted for sex (male or female), study center (Seoul or Suwon), and baseline age (continuous).

§ Further adjusted for height (continuous) and baseline levels of intraocular pressure measurement time (morning or afternoon), smoking status (never, former, or current), alcohol drinking (< 1, 1–3, or > 3 days/week), physical activity (none, 1–3, or > 3 times/week), and heart rate (continuous).

|| Further adjusted for baseline hypertension (yes or no) and diabetes (yes or no).

**Fig 3:**
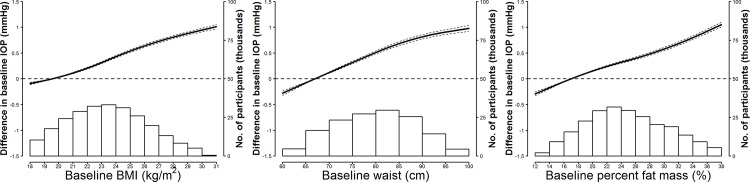
Smooth longitudinal associations between changes in adiposity markers and intraocular pressure over time. Curves represent average longitudinal changes in intraocular pressure (solid lines) and their 95% confidence intervals (dashed lines) based on restricted quadratic splines for adiposity marker changes over time with knots at the 5th, 50th, and 95th percentiles and constrained to be 0 at baseline. Results were obtained from linear mixed models with random variations in baseline intraocular pressure levels and intraocular pressure changes over time across participants and between eyes within participants, and adjusted for baseline adiposity marker levels (restricted quadratic splines), sex (male or female), study center (Seoul or Suwon), height (continuous), and baseline levels and changes over time in age (continuous), intraocular pressure measurement time (morning or afternoon), smoking status (never, former, or current), alcohol drinking (< 1, 1–3, or > 3 days/week), physical activity (none, 1–3, or > 3 times/week), heart rate (continuous), hypertension (yes or no), and diabetes (yes or no). Histograms represent the frequency distributions of adiposity marker changes.

**Fig 4:**
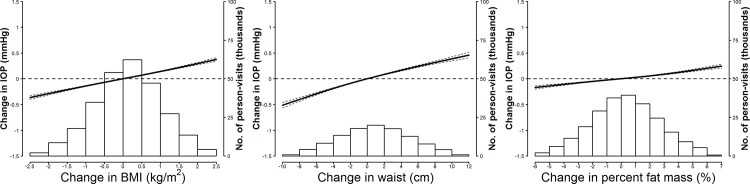
Smooth longitudinal associations between changes in adiposity markers and intraocular pressure over time, by sex. Curves represent average longitudinal changes in intraocular pressure (solid lines) and their 95% confidence intervals (dashed lines) based on restricted quadratic splines for adiposity marker changes over time with knots at the 5th, 50th, and 95th percentiles and constrained to be 0 at baseline. Results were obtained from linear mixed models with interactions between spline terms and sex, random variations in baseline intraocular pressure levels and intraocular pressure changes over time across participants and between eyes within participants, and adjusted for baseline adiposity marker levels (restricted quadratic splines), study center (Seoul or Suwon), height (continuous), and baseline levels and changes over time in age (continuous), intraocular pressure measurement time (morning or afternoon), smoking status (never, former, or current), alcohol drinking (< 1, 1–3, or > 3 days/week), physical activity (none, 1–3, or > 3 times/week), heart rate (continuous), hypertension (yes or no), and diabetes (yes or no).

In longitudinal analyses, increases in adiposity markers over time were associated with changes in IOP (**Table 3**). In models adjusted for potential confounders (Model 2), the average IOP increase associated with an interquartile increase in BMI, waist circumference, and percent fat mass was 0.181 mmHg (95% confidence interval [CI] 0.172 to 0.191), 0.270 mmHg (0.255 to 0.285) and 0.103 mmHg (0.093 to 0.113), respectively. The positive longitudinal associations in IOP with change in adiposity markers were also evident in restricted quadratic spline models, which showed a stronger association for BMI and waist circumference compared to percent fat mass (**Fig 3**). Adjusting for hypertension and diabetes as potential mediators (Model 3) had only a small impact on the associations. The longitudinal associations between adiposity and IOP were significantly stronger in men than in women (**Fig 4**). Sensitivity analyses without excluding participants with abnormal fundoscopy findings or with between-eye differences in IOP greater than 6 mmHg and restricting the study population to participants with at least two screening visits also did not affect the results (data not shown).

**Table 3:** Longitudinal associations of changes in adiposity markers with changes in intraocular pressure over time.*

	Interquartile increase in adiposity marker over time	Quartile of change in adiposity marker				
		Quartile 1	Quartile 2	Quartile 3	Quartile 4	*P* value†
**Body mass index**						
Median change (range), kg/m^2^	0.14 (-11.35, 11.76)	-1.00 (-11.35, -0.49)	-0.15 (-0.49, 0.14)	0.43 (0.14, 0.77)	1.26 (0.77, 11.76)	
No. of subjects/visits	130,855/303,749	45,881/75,937	54,132/75,938	54,195/75,937	45,363/75,937	
Adjusted mean change in intraocular pressure (95% CI), mmHg						
Unadjusted	0.179 (0.170, 0.188)	-0.393 (-0.408, -0.378)	-0.218 (-0.232, -0.205)	-0.128 (-0.142, -0.115)	0.004 (-0.011, 0.019)	< 0.001
Model 1‡	0.203 (0.193, 0.212)	-0.255 (-0.272, -0.238)	-0.097 (-0.113, -0.081)	-0.007 (-0.022, 0.009)	0.145 (0.127, 0.163)	< 0.001
Model 2§	0.181 (0.172, 0.191)	-0.214 (-0.232, -0.197)	-0.079 (-0.095, -0.062)	0.004 (-0.012, 0.020)	0.138 (0.120, 0.157)	< 0.001
Model 3||	0.181 (0.171, 0.190)	-0.212 (-0.230, -0.195)	-0.075 (-0.091, -0.059)	0.007 (-0.009, 0.023)	0.140 (0.122, 0.158)	< 0.001
**Waist circumference**						
Median change (range), cm	0.80 (-54.80, 40.20)	-4.50 (-54.80, -2.30)	-0.70 (-2.30, 0.80)	2.20 (0.80, 3.90)	6.10 (3.90, 40.20)	
No. of subjects/visits	59,459/120,850	20,431/30,392	23,123/30,142	23,139/30,003	20,619/30,313	
Adjusted mean change in intraocular pressure (95% CI), mmHg						
Unadjusted	0.238 (0.223, 0.253)	-0.537 (-0.560, -0.514)	-0.324 (-0.345, -0.302)	-0.190 (-0.211, -0.169)	-0.051 (-0.073, -0.030)	< 0.001
Model 1‡	0.286 (0.271, 0.301)	-0.381 (-0.408, -0.354)	-0.171 (-0.197, -0.146)	-0.037 (-0.062, -0.011)	0.120 (0.093, 0.146)	< 0.001
Model 2§	0.270 (0.255, 0.285)	-0.354 (-0.382, -0.327)	-0.160 (-0.186, -0.134)	-0.038 (-0.064, -0.012)	0.117 (0.090, 0.144)	< 0.001
Model 3||	0.269 (0.253, 0.284)	-0.352 (-0.380, -0.324)	-0.159 (-0.185, -0.133)	-0.037 (-0.063, -0.011)	0.118 (0.091, 0.145)	< 0.001
**Percent fat mass**						
Median change (range), %	0.29 (-60.47, 31.56)	-2.70 (-60.47, -1.39)	-0.49 (-1.39, 0.29)	1.08 (0.29, 2.00)	3.35 (2.00, 31.56)	
No. of subjects/visits	117,867/261,475	42,564/65,369	47,794/65,369	47,721/65,368	42,358/65,369	
Adjusted mean change in intraocular pressure (95% CI), mmHg						
Unadjusted	0.092 (0.083, 0.102)	-0.240 (-0.256, -0.224)	-0.152 (-0.166, -0.137)	-0.112 (-0.126, -0.097)	-0.036 (-0.051, -0.020)	< 0.001
Model 1‡	0.126 (0.117, 0.136)	-0.165 (-0.183, -0.147)	-0.080 (-0.097, -0.063)	-0.032 (-0.049, -0.014)	0.080 (0.061, 0.099)	< 0.001
Model 2§	0.103 (0.093, 0.113)	-0.122 (-0.141, -0.103)	-0.058 (-0.076, -0.040)	-0.021 (-0.039, -0.003)	0.072 (0.053, 0.092)	< 0.001
Model 3||	0.100 (0.090, 0.110)	-0.117 (-0.136, -0.098)	-0.054 (-0.071, -0.036)	-0.018 (-0.036, -0.001)	0.073 (0.053, 0.092)	< 0.001

* Results were obtained from linear mixed models with random variations in baseline intraocular pressure levels and intraocular pressure changes over time across participants and between eyes within participants.

† *P* value for linear trend using an ordinal variable with the median adiposity marker change in each quartile.

‡ Adjusted for baseline adiposity marker levels (either continuous or quartiles), sex (male or female), study center (Seoul or Suwon), baseline age (continuous), and age change over time (continuous).

§ Further adjusted for height (continuous) and baseline levels and changes over time in intraocular pressure measurement time (morning or afternoon), smoking status (never, former, or current), alcohol drinking (< 1, 1–3, or > 3 days/week), physical activity (none, 1–3, or > 3 times/week), and heart rate (continuous).

|| Further adjusted for baseline levels and changes over time in hypertension (yes or no) and diabetes (yes or no).

## Discussion

In this large cohort of Korean adults, increases over time in BMI, waist circumference and percent fat mass were positively associated with longitudinal increases in IOP. The associations were strongest for waist circumference, and were stronger in men than in women. These associations persisted after adjusting for hypertension and diabetes, suggesting that adiposity may affect IOP through pathways that are independent from blood pressure or glucose control. The strengths of our study included the large sample size, the longitudinal approach, the availability of waist circumference and percent fat mass in addition to BMI, the availability of repeated data on numerous potential confounders, and the use of statistical methods that appropriately accounted for the correlations between IOP measured in both eyes in each participants and across visits for each participant.

Previous longitudinal studies also found a positive association between BMI and IOP,[12, 23–28] but the Beijing Eye study[25] and a Japanese study[12] had only two measurements 5 years apart, while three other studies in Japan[24, 26, 28]and the Barbados Eye Study[27] did not provide detailed dose-response relationships. Furthermore, our study was the only one providing information on waist circumference and percent fat mass in addition to BMI as adiposity markers. Indeed, in our study, waist circumference was the marker showing the strongest association with IOP among adiposity markers, suggesting that central adiposity plays a role in determining IOP levels.

Several mechanisms may explain an increase in IOP with higher adiposity. First, increased adiposity is associated with increased oxidative stress, which may result in trabecular meshwork degeneration, may impair the ability of the intracellular system to modulate outflow resistance, and may lead to increased IOP.[35] Increased adiposity may also induce vascular endothelial dysfunction and autonomic dysfunction,[36, 37] which are associated with abnormal ocular blood flow and perfusion instability. Second, increased adiposity is a major cause of hypertension and diabetes, which are associated with increased IOP and glaucoma.[14, 19] Increased blood pressure increases filtration of aqueous humor through elevated capillary pressure and reduces aqueous humor outflow through elevated episcleral venous pressure.[38] Hyperglycemia may induce an osmotic gradient that shifts excess aqueous humor into the anterior chamber.[39] In our analysis, the association between adiposity markers and IOP was only slightly attenuated (change in IOP associated with an interquartile increase 0.181 vs 0.181, 0.269 vs 0.270, and 0.100 vs 0.103 for BMI, waist circumference, and percent fat mass, respectively) after we adjusted for hypertension and diabetes, suggesting that other mechanisms may link excess adiposity and increased IOP. Finally, adiposity markers may partially reflect intraobital adipose tissue. Excess intraorbital adipose tissue may increase episcleral venous pressure and impair aqueous humor outflow, resulting in increased IOP.[40, 41]

In our study, the longitudinal association of adiposity markers with IOP was stronger in men compared with women. The increase in IOP with age in this cohort was also stronger in men compared with women, irrespective of changes in BMI.[10] There is no clear explanation for these gender differences. Men have a higher volume of visceral abdominal adipose tissue than women,[42] although the association was stronger in men for both general and visceral adiposity markers. Hormonal differences may explain the heterogeneity by sex.[43] Estrogen levels, which increase with increasing adiposity, may affect the inflow of aqueous humour, the ciliary body and the trabecular meshwork,[44, 45] but further research is needed to understand gender-related differences in adiposity and IOP.

Our findings suggest that increased adiposity may be a minor contributing factor to increased IOP, and may increase the risk of glaucoma or complicate the control of IOP in glaucoma. The effect sizes associated with an interquartile range increase in adiposity markers were small, which may limit the clinical impact on glaucoma treatment. However, our study only captured a limited follow-up span (average follow-up among participants with at least 2 visits 3.6 years), and the increase in adiposity can be large with longer follow-up times. In addition, there was wide within-person variability, and it is possible that a subgroup of participants are particularly sensitive to the IOP-rising effects of adiposity. The effect of weight loss on IOP reduction and glaucoma prognosis should also be evaluated in patients with glaucoma as it may complement other strategies for IOP reduction and disease management. These results may also imply potential new pathways for research on glaucoma treatment and control.

Some limitations of our study also need to be considered. First, we used non-contact tonometers to measure IOP instead of applanation tonometers, considered the gold standard. Although non-contact tonometers are as reliable as applanation tonometers,[46] use of non-contact tonometers may have resulted in measurement error that underestimated the study associations. Second, our study included preferentially young and middle-age Korean men and women attending health screening visits, which may limit the generalizability of our findings to other study populations. Third, we could not assess the performance of the individual examiner and we could not fully exclude the possibility of observer bias, but this type of bias may be less relevant in longitudinal studies. In addition, examiners were not aware of the study hypotheses.

In conclusion, we found that excess adiposity was associated with statistically significantly increased IOP in a large cohort of Korean adults attending health screening visits, an association that was stronger for central obesity. Further research is needed to better understand the underlying mechanisms of this association, and to establish the role of weight reduction in reducing IOP and delaying or preventing glaucoma complications.

## Statistical Appendix

### Cross-sectional and longitudinal associations of adiposity markers with intraocular pressure

To assess the cross-sectional association of body adiposity markers (body mass index, waist circumference, and percent fat mass) with intraocular pressure (IOP) at baseline, as well as the longitudinal association between changes in adiposity markers and IOP over time, we developed linear mixed models for longitudinal paired-eye data using a three-level hierarchical approach.[33, 47] At the first within-eye level, the change in IOP levels *Y*_*ijt*_ from baseline visit *t* = 0 to follow-up visits *t* = 1,…, *m*_*i*_ for eye *j* = 1, 2 of participant *i* = 1,…, *n* was related to the corresponding changes in the adiposity marker *x*_*it*_ and other subject-specific time-varying covariates **z**_*it*_ through the linear model

$$Y_{ijt}=\;\alpha_{ij0}+\;\alpha_{ij1}\;(x_{it}-x_{i0})+{\alpha^{,}\prime }_{ij2}(z_{it}-\;z_{i0})+\;\varepsilon_{ijt},$$

where *α*_*ij0*_ was the expected baseline IOP for eye *j* of participant *i*; *α*_*ij1*_ was the expected change in IOP over time for eye *j* of participant *i* per unit change in the adiposity marker; ***α***_*ij2*_ were the expected longitudinal IOP slopes for that eye associated with the other time-varying covariates, including age (continuous), IOP measurement time (morning or afternoon), smoking status (never, former, or current), alcohol drinking (< 1, 1–3, or > 3 days/week), physical activity (none, 1–3, or > 3 times/week), heart rate (continuous), hypertension (yes or no), and diabetes (yes or no); and the within-eye errors *ε*_*ijt*_ were assumed to be independent and normally distributed with mean 0 and constant variance *σ*^2^.

The second level represented the variation in coefficients *α*_*ij0*_, *α*_*ij1*_, and ***α***_*ij2*_ between eyes of the same participant. The eye-specific baseline IOP *α*_*ij0*_ was allowed to vary randomly between each participant’s eyes, whereas the longitudinal IOP slopes for the adiposity marker *α*_*ij1*_ and the other time-varying covariates ***α***_*ij2*_ were assumed to be fixed for both eyes. Thus, the second-level model was

$$\alpha_{ij0}=\;\gamma_{i00}\;+\;b_{ji0},$$

$$\alpha_{ij1}=\;\gamma_{i10},$$

$$\alpha_{ij2}=\;\gamma_{i20},$$

where the between-eye variations within a subject *b*_*ij0*_ were assumed to be independent and normally distributed with mean 0 and constant variance *τ*^2^. The longitudinal slopes associated with body adiposity markers *α*_*ij1*_ were specified as fixed at eye level because preliminary analyses showed virtually null random variation in longitudinal IOP slopes between each participant’s eyes.

Finally, the third level described the variation in parameters *γ*_*i00*_, *γ*_*i10*_, and ***γ***_*i20*_ across participants. The subject-specific baseline IOP *γ*_*i00*_ was linearly related to each participant’s baseline levels of the adiposity marker *x*_*i0*_ and time-varying covariates **z**_*i0*_, as well as to other time-constant covariates **w**_*i*_, including sex (female or male), study center (Seoul or Suwon), and height (continuous). The subject-specific longitudinal IOP slopes for the adiposity marker *γ*_*i10*_ were allowed to vary randomly across participants, while the longitudinal slopes for the other time-varying covariates ***γ***_*i20*_ were assumed to be constant for all participants. Specifically, the third-level model was

$$\gamma_{i00}=\;\beta_{000}+\;\beta_{001}x_{i0}+\beta_{002}z_{i0}+\beta_{003}w_{i}+b_{i00},$$

$$\gamma_{i10}=\;\beta_{100}+\;b_{i10},$$

$$\gamma_{i20}=\;\beta_{200},$$

where the between-subject variations *b*_*i00*_ and *b*_*i10*_ were assumed to follow a bivariate normal distribution with mean **0** and constant variance-covariance matrix **V**. Combining the three nested models, we obtained the linear mixed model

$$Y_{ijt}=\left(\beta_{000}+b_{i00}+b_{ji0}\right)+\;\beta_{001}x_{i0}+\beta_{002}z_{i0}+\beta_{003}w_{i}+\left(\beta_{100}+\;b_{i10}\right)(x_{it}-x_{i0})+\beta_{200}(z_{it}-\;z_{i0})+\varepsilon_{ijt},$$

which included two nested random effects for the intercept, as well as a random effect at subject level for the longitudinal slope associated with the adiposity marker, to account for correlations arising from both paired-eye data and repeated measurements over time.[34]

In this mixed model, the first line on the right-hand side represented the cross-sectional association of the adiposity marker with IOP at baseline. In particular, the fixed effect *β*_001_ corresponded to the mean difference in baseline IOP per unit increase in the baseline adiposity marker adjusted for baseline covariates. To allow for nonlinear cross-sectional relationships, we modified the fixed-effects specification for *x*_*i0*_ to compare the mean baseline IOP across quartiles of the baseline adiposity marker, as well as to estimate smooth baseline IOP changes as a restricted quadratic spline function of the baseline adiposity marker with knots at the 5th, 50th, and 95th percentiles.[48] Also, to evaluate potential heterogeneity of cross-sectional associations by sex, we included fixed-effects interactions between sex and restricted quadratic spline terms for the baseline adiposity marker.

The second line of the above linear mixed model represented the longitudinal association of within-subject changes in the adiposity marker and IOP over time.[49] Specifically, the fixed effect *β*_100_ corresponded to the mean change in IOP over time per unit increase in the within-subject adiposity marker adjusted for baseline and time-varying covariates. To assess nonlinear longitudinal effects, we alternately replaced the linear term *x*_*it*_ − *x*_*i0*_ in the above model with quartile indicators and restricted quadratic splines for within-subject changes in the adiposity marker with knots at the 5th, 50th, and 95th percentiles and constrained to be 0 at baseline. Quartile indicators and linear spline terms were specified as random at subject level to allow for random between-subject variations around the average nonlinear longitudinal trend. In addition, longitudinal effect modifications by sex were explored by further including fixed-effects interactions between sex and restricted quadratic spline terms for adiposity marker changes.
